# Synthesis and Characterization of Silver and Gold Nanoparticles Using Aqueous Extract of Seaweed, *Turbinaria conoides,* and Their Antimicrofouling Activity

**DOI:** 10.1155/2014/938272

**Published:** 2014-02-03

**Authors:** Sri Ramkumar Vijayan, Prakash Santhiyagu, Muthukkumarasamy Singamuthu, Natarajan Kumari Ahila, Ravindran Jayaraman, Kannapiran Ethiraj

**Affiliations:** ^1^Department of Oceanography and Coastal Area Studies, School of Marine Sciences, Alagappa University, Thondi Campus, Thondi, Tamil Nadu 623 409, India; ^2^SRM Research Institute, SRM University, Kattankulathur, Kancheepuram, Tamil Nadu 603 203, India; ^3^Department of Nanoscience and Technology, Alagappa University, Karaikudi, Tamil Nadu 630 003, India; ^4^Department of Zoology, Directorate of Distance Education, Alagappa University, Karaikudi, Tamil Nadu 630 003, India; ^5^Biological Oceanography Division, CSIR-National Institute of Oceanography, Dona Paula, Goa 403 004, India

## Abstract

Silver and gold nanoparticles were synthesized using an aqueous extract of the seaweed *Turbinaria conoides* and their antibiofilm activity against marine biofilm forming bacteria is reported here. The UV-Vis spectra showed the characteristics SPR absorption band for Ag NPs at 421 and for Au NPs at 538 nm. Further, the synthesized nanoparticles were characterized using FT-IR, XRD, FESEM, EDX, and HRTEM analysis. Spherical and triangular nanostructures of the Ag and Au nanoparticles were observed between the size ranges of 2–17 nm and 2–19 nm, respectively. The synthesized Ag NPs are efficient in controlling the bacterial biofilm formation; however, Au NPs did not show any remarkable antibiofilm activity. The maximum zone of inhibition was recorded against *E. coli* (17.6 ± 0.42 mm), followed by *Salmonella* sp., *S. liquefaciens*, and *A. hydrophila*. The macrotube dilution method inferred the MIC (20–40 *µ*L mL^−1^) and MBC (40–60 *µ*L mL^−1^) of Ag NPs. The CLSM images clearly showed the weak adherence and disintegrating biofilm formation of marine biofilm bacterial strains treated with Ag NPs. The *Artemia* cytotoxicity assay recorded the LC_50_ value of 88.914 ± 5.04 *µ*L mL^−1^. Thus the present study proved the efficiency of Ag NPs as a potent antimicrofouling agent and became the future perspective for the possible usage in the biofouling related issues in the aquaculture installations and other marine systems.

## 1. Introduction

Natural and artificial substrata immersed in the aquatic environment are quickly colonized by micro- and macroorganisms; this phenomenon is known as “Biofouling” [1]. Biofouling is a sequence of processes initialized by the attachment of microbes to a solid support by producing extracellular polymeric substances and thus promoting the development of a biofilm matrix. It depends upon an interaction between the bacterial cells, surface attachment, and surrounding medium [2–5]. The biofilm formation further favours the attachment of other macrofoulers [6, 7]. These biofouling processes affect marine installations such as ship's hull, oil rigs, mariculture cages, underwater pipelines, heat exchangers, and seawater intake systems [8, 9]. It possesses serious problems, such as corrosion, weight increase, surface alteration and distortion of the submerged structures, and speed reduction and increased fuel consumption up to 40% [10], which also contributes to additional CO_2_ emissions. Thus, the economic loss is stupendous in preventing and removing the fouling organism.

Numerous antibiofouling measures such as mechanical, chemical, and biological methods are in practice but their effects on the biofouling are not remarkable. In addition, the commercially available antifouling paints such as tributyltin (TBT) and copper sulphate are highly toxic to the nontarget aquatic organisms [11]. The TBT contributes to the development of antimicrobial tolerance and imposex or pseudohermaphroditism in marine invertebrates [12].

Application of antifouling compounds from natural sources is considered as one of the best replacement options for the most successful antifouling processes [13]. A wide variety of marine natural products from seagrasses, seaweeds, mangroves, coral reefs, and their associated organisms proved to be an excellent source of bioactive compounds and a wide range of secondary metabolites, many of which exhibited a broad spectrum of antifouling activity against marine biofilm forming microbes, algal spore adherence, mussel (phenoloxidase activity), and barnacles to artificial substratum [14–16].

Comparing the above antifoulers, “nano materials” are efficient in inhibiting the bacterial adhesion and biofilm formation due to their effective antimicrobial property and large specific surface area, which is inversely proportional to their particle size. Silver, copper, zinc, and magnesium fluoride nanoparticles were having good antimicrobial properties and also reduced the cell adhesion and destabilized the biofilm matrix [17, 18]. Earlier reports demonstrated that ZnO, CuO, and Ag nanoparticles (NPs) coated on solid surfaces inhibit the biofilm formation [19, 20] and particularly Ag NPs act as potential antimicrobial agents against different microorganisms [21, 22]. There are several methods to synthesize the nanoparticles, but the greener method is more advantageous as it is cost effective, energy efficient, and does not involve the use of more chemicals, thus is an ecofriendly and reliable method [23].

Seaweeds are macrophytic marine algae that produce a great variety of secondary metabolites having broad spectrum of biological activities. So far, several researchers reported the synthesis of nanoparticles using seaweeds [24, 25] and their biological applications [26–29]. However, reports on the marine antimicrofouling property of biologically synthesized silver and gold nanoparticles are scanty. Hence, the present study was carried out to understand the antibiofilm activity against marine biofilm forming bacteria and *Artemia* cytotoxicity of biosynthesized silver nanoparticles (Ag NPs) and gold nanoparticles (Au NPs) using the aqueous extract of seaweed *Turbinaria conoides*.

## 2. Materials and Methods

### 2.1. Chemicals

Silver nitrate (AgNO_3_; Merck, India) and chloroauric acid (HAuCl_4_·3H_2_O; Sigma Aldrich, USA) were used in this study. All the microbiological media and chemicals were obtained from HiMedia Laboratories, India. Ultrapure Milli Q water was used throughout the study.

### 2.2. Collection of Seaweed

The brown seaweeds, *Turbinaria conoides* (J. Agardh, Kutzing, 1860), were collected from Mandapam coastal region (78°8′E, 9°17′N), Gulf of Mannar, Southeast coast of India. They were washed thoroughly with running tap water followed by distilled water to remove adhering salts and associated biota. The washed samples were dried under shade at room temperature for a week. The dried materials were ground to fine powder using mixer grinder.

### 2.3. Preparation of Aqueous Extract and Synthesis of Ag and Au NPs

5 g of the seaweed powder was boiled with 100 mL of sterile deionised water for 10–15 min and filtered through Whatman number 1 filter paper. The resultant aqueous filtrate was treated individually with 90 mL aqueous solution of (1 mM) AgNO_3_ and HAuCl_4_·3H_2_O. The mixture was incubated at room temperature and the color change in the reaction solution was noted by visual observation.

### 2.4. Characterization of Ag and Au NPs

Surface plasmon resonance (SPR) absorption bands were measured using a UV-Visible spectrophotometer (UV-1800 Shimadzu, Japan) by scanning operated at a resolution of 1 nm, between 200 and 800 nm. The crystal structure of the Ag and Au NPs was characterized by the X-ray diffraction (XRD) pattern using X'Pert PRO analytical X-ray diffractometer operating at a voltage of 45 kV in the current of 40 mA using Cu K*α* radiation with X'Pert High Score Plus Software. The Fourier Transform Infrared (FT-IR-Nicolet Thermo spectrophotometer iS5, USA) spectra of seaweed extract and synthesized Ag and Au NPs colloidal solutions were analyzed in the range between 4000 and 400 cm^−1^ with a resolution of 4 cm^−1^ to arrive at the possible functional groups responsible for reduction and capping behaviour of biomolecules present in the seaweed extracts. A drop of colloidal nanoparticles were coated on a copper plate, dried in a hot air oven, and examined using Field Emission Scanning Electron Microscopy (FESEM) (Carl Zeiss, Germany) equipped with Energy Dispersive X-ray spectroscopy (EDX-JEOL, JSM-5610) analysis. Morphology and size of the nanoparticles were further authenticated by the HRTEM image. A drop of Ag and Au NPs were coated on a carbon coated copper grid of 200 mesh size and dried for 5 min prior to the observation in a High Resolution Transmission Electron Microscope (HRTEM) (JEOL JEM 2100 HRTEM) operated at an accelerating voltage of 200 kV. The selected area electron diffraction (SAED) pattern was also performed.

### 2.5. Antibacterial and Antibiofilm Assay against Marine Biofilm Bacterial Strains

#### 2.5.1. Selection of Marine Biofilm Forming Bacterial Strains

Four different marine biofilm forming bacterial strains, namely, *Salmonella* sp. (JN596113), *E. coli* (JN585664), *S. liquefaciens* (JN596115), and *A. hydrophila* (JN561697) isolated by Prakash (2012, unpublished data) from different substratum and immersed in Thondi coastal waters, Palk Bay, Southeast coast of India, were used to study the antibiofilm activity of the synthesized Ag and Au NPs.

#### 2.5.2. Antibacterial Potential of Ag and Au NPs

Antibacterial activity of the synthesized Ag and Au NPs screened against marine biofilm bacterial strains using agar well diffusion method [30, 31]. In brief, all the biofilm bacterial strains were individually inoculated in the Zobell marine broth and incubated for 12 h. The biofilm bacterial cells (~1 × 10^8^ cells mL^−1^) were inoculated using sterile cotton swabs on the Mueller Hinton Agar plates prepared with 50% seawater. Experimental wells of 6 mm diameters on the plates were loaded with 50 *μ*L of colloidal nanoparticles; the sterilized distilled water was used as a negative control and incubated for 24 h at 37°C. The assay was carried out in triplicates. The zone of inhibition was measured from the well to the edge of the clear zone in millimetre (mm).

#### 2.5.3. Minimum Inhibitory Concentration (MIC) and Minimum Bactericidal Concentration (MBC) of NPs

The MIC and MBC of the NPs were determined according to the method of Ruparelia et al. [31] with some modification. Forty microlitres (~1 × 10^8^ cells mL^−1^) of each biofilm forming bacterial strains were added individually to 4 mL of Zobell marine broth (ZMB). The different concentrations (5, 10, 20, 40, 60, 80, and 100 *μ*L) of the NPs solutions were added to the test tubes containing the test strains. After 24 h of incubation, the MIC results were noted by checking the turbidity of the bacterial growth and the MBC were determined by streaking a loop full of the bacterial culture on the Zobell marine Agar (ZMA) plates, incubated at 37°C for 24 h. The MBC is the concentration at which the bacteria are completely killed.

#### 2.5.4. Confocal Laser Scanning Microscopic (CLSM) Analysis

The effect of biosynthesized Ag and Au NPs on the adherence and biofilm formation were observed under a Confocal Laser Scanning Microscope (Carl Zeiss, LSM 710). In concise, the presterilized glass slides of 1 cm × 1 cm each were placed in the 24-well polystyrene plates containing (total volume —2.5 mL) 2.3 mL of sterilized Zobell marine broth, 60 *μ*L of Ag NPs, and 100 *μ*L of test biofilm bacterial strains (~1.5 × 10^8^ cells mL^−1^), incubated at 37°C. The respective strains without the NPs were considered as a negative control. After 24 h of incubation, the glass slides were taken carefully and washed with PBS (Phosphate Buffer Solution) to remove the loosely attached bacterial cells. Each glass slide was stained with 0.1% acridine orange and examined in the CLSM at a magnification of 63x. The 488 nm argon laser and a 500–640 nm band pass emission filter were used to excite and detect the stained cells. The Z-stack analysis (surface topography and 3D architecture) was calculated using Zen 2009 software [32].

### 2.6. Cytotoxicity and Anticrustacean Assay

Cytotoxicity assay is one of the easiest screening assays to test the toxicity of bioactive materials. Here, we used *Artemia salina *as the test organisms. It is one of the major marine biofouling organism belonging to Crustaceous. The larvae (I instar) of *A. salina* hatched out as described by Harwig and Scott [33]. For the toxicity test, varying concentrations of 40, 60, 80, 100, 120, 140, and 160 *μ*L mL^−1^ of the NPs solution were transferred to the 24-well plates containing 1 mL of sterilized seawater [34]. The live nauplii of *A. salina* (10 nauplii per well) were added to it and incubated at 25°C. Seawater without the NPs was the control. After 24 h of incubation, surviving *A. salina *larvae were counted in each well.

### 2.7. Statistical Analysis

The data generated were expressed as a mean ± SD. The analysis of variance (one-way ANOVA; *P* < 0.05) was performed for the mean value of antibacterial properties of Ag NPs against marine biofilm bacterial strains. The percentage mortality of *A. salina* larvae in the different test concentrations of the Ag NPs and the negative control was compared by Dunnett test with varied significant levels (*P* < 0.0001; *P* < 0.01). All the analyses were carried out using SPSS v16.0 (SPSS Inc., USA). Profit analysis of the toxicity data was calculated with EPA profit analysis software ver.1.5, USA.

## 3. Results and Discussion

### 3.1. UV-Visible Spectrum

**Figure 1:**
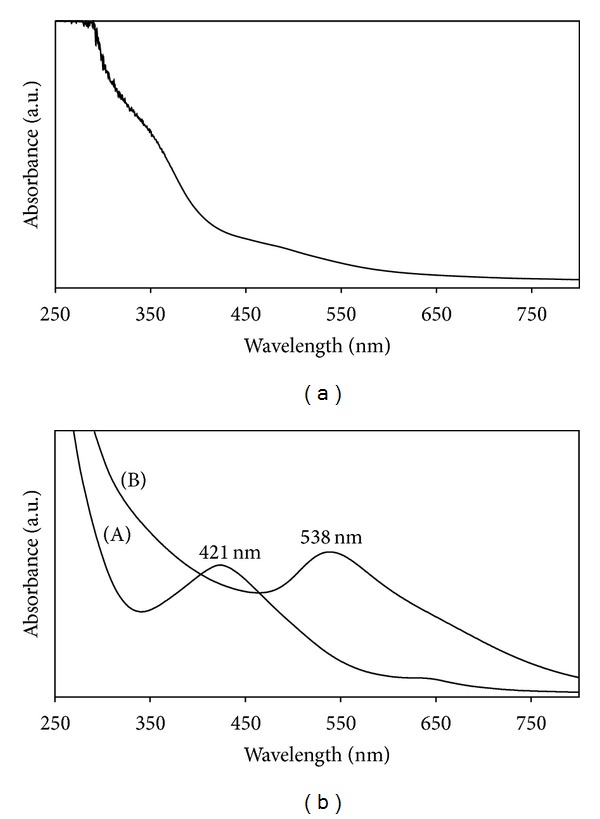
(a) UV-Vis spectrum of *T. conoides* extract; (b) synthesized colloidal Ag NPs (A) and Au NPs (B).

The UV-Vis spectrum observed at 421 nm and 538 nm confirmed the synthesis of Ag and Au NPs, respectively (Figure 1(b) (A and B)). The UV-Vis spectrum of seaweed *T. conoides* aqueous extract has shown no significant peaks (Figure 1(a)). After the addition of *T. conoides *extract with the colourless silver nitrate and yellow coloured chloroauric acid the solutions were changed to characteristic reddish brown and ruby-red colour provided a convenient excitation of SPR, which indicates the formation of Ag and Au NPs [35, 36]. The present study also observed the rapid synthesis of Ag and Au NPs within 15–20 and 10–15 minutes, respectively.

### 3.2. X-Ray Diffraction Patterns

**Figure 2:**
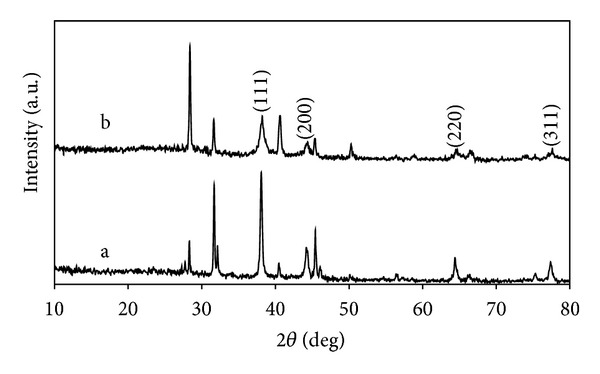
XRD patterns of Ag NPs (a) and Au NPs (b).

The X-ray diffraction patterns of the Ag and Au NPs synthesized using *T. conoides *extract are shown in Figures 2(a) and 2(b). Presence of four distinct high diffraction peaks at 38.1°, 44.2°, 64.3°, and 77.3°, respectively, indexing the Bragg's reflection planes (111), (200), (220), and (311) (JCPDS No. 04-0783) confirmed the face-centered cubic silver. Similarly, the XRD pattern of the Au NPs showed a strong diffraction peak at 38.1° attributed to (111) facet of the face-centered cubic metal gold structure. The other three diffraction peaks (44.3°, 64.5°, and 77.5° for the facets of (200), (220), and (311) (JCPDS File number 04-0784)) were also detected. Thus, the XRD results revealed that the Ag and Au NPs were formed by the reduction of the metal ions by *T. conoides* extract was crystalline in nature. Similar structures of the Ag and Au NPs were synthesized using terrestrial plants reported earlier by Chandran et al. [37] and Philip and Unni [38].

### 3.3. Fourier Transform Infrared Spectrum

**Figure 3:**
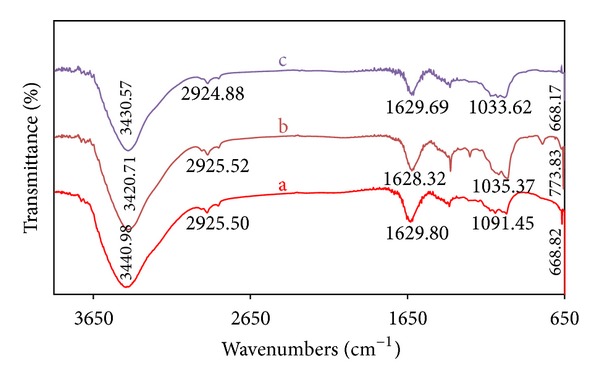
FT-IR spectra of *T. conoides* extract (a), Ag NPs (b), and Au NPs (c).

The FT-IR spectrum of *T. conoides* extract, the colloidal Ag NPs and Au NPs, envisages that the molecular arrangement of different functional groups (Figures 3(a)–3(c)). When all the three transmission peaks were compared, the suppressed/increased peaks in the colloidal solutions were due to the consequence of metal nanoparticles bound to bioorganic molecules. The shifted peaks such as 3441 and 1629 cm^−1^ were characteristics of O–H and C=O stretching modes due to a free hydroxyl group and a carboxylic acid group, respectively. Another band at 1020 cm^−1^ could be assigned to the C–OH vibrations of primary alcohol groups in the extracts. It is evident through the FT-IR spectra that the presence of different functional groups in the aqueous seaweed extract might serve as the reducing and capping agents of the Ag and Au NPs. Similar phenomenon was also observed by some authors to identify the possible biomolecules responsible for the reduction and capping of the Ag and Au NPs synthesized using other seaweeds [39].

### 3.4. Field Emission Scanning Electron Microscopic Analysis

**Figure 4:**
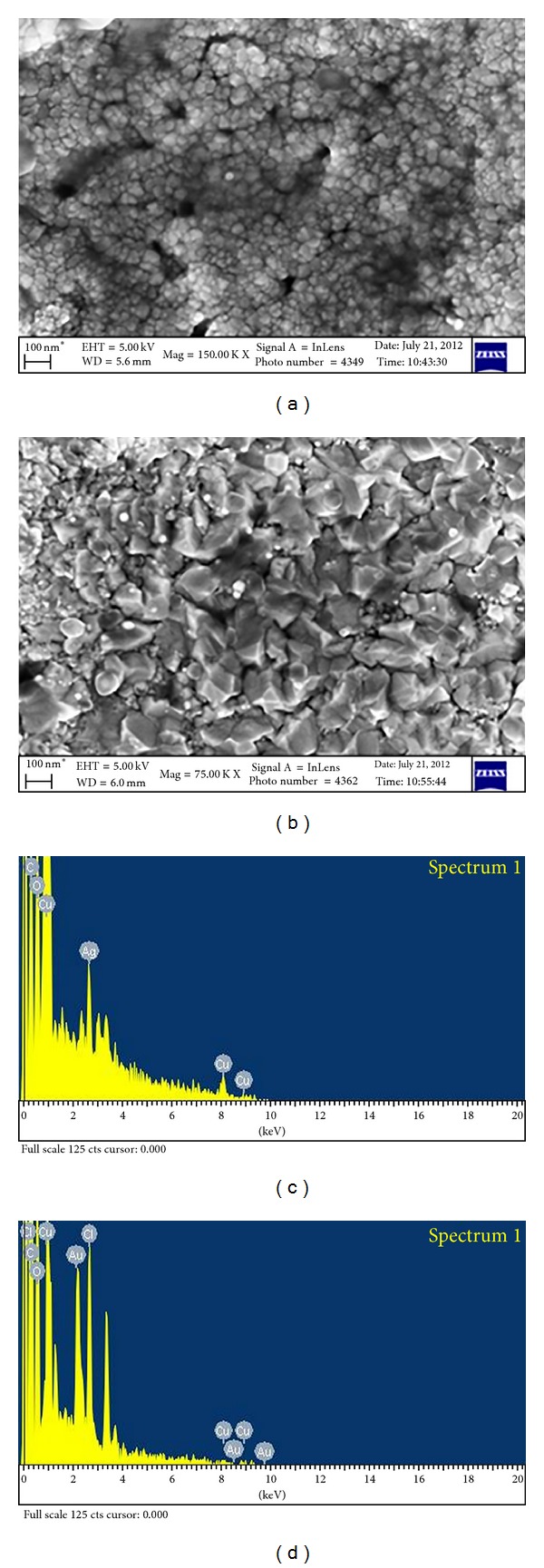
FESEM image of Ag (a) and Au NPs (b) with EDX graph of Ag (c) and Au (d).

The FESEM images distinguished the spherical and triangular morphology of the Ag and Au NPs with the size less than 60 nm (Figures 4(a) and 4(b)). Size distribution analysis of the capped Ag and Au nanoconjugates confirmed that the particles were well dispersed. The qualitative and quantitative analysis by the EDX revealed the highest proportion of Ag and Au signals (Figures 4(c) and 4(d)). Minor peaks of the copper and carbon were observed that could be the biomolecules bound to the surface of the NPs and the copper used for coating the samples.

### 3.5. High Resolution Transmission Electron Microscopic Studies

**Figure 5:**
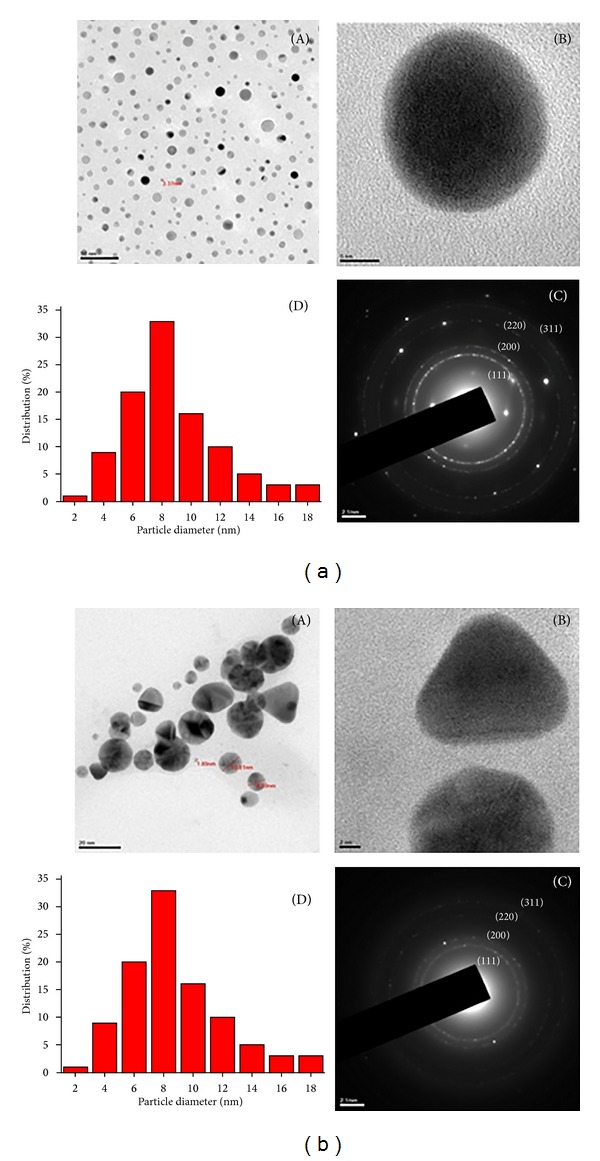
HRTEM images of Ag NPs (a) and Au NPs (b); different magnifications (A) and (B); SAED pattern (C); particle size distribution (D).

In addition, the HRTEM provided further insight into the morphology and sizes of the Ag and Au NPs. The NPs were well dispersed and no agglomeration was noticed. The uniformly small sized spherical and fascinating triangular shaped nanoparticles of the sizes ranging between 2–17 nm and 2–19 nm of the Ag NPs and Au NPs (Figures 5(a) and 5(b)), respectively, were microscopically visualized. These narrow ranged sizes of nanoparticles agreed with the SPR results. The crystalline nature of Ag and Au NPs was evidenced by the SAED pattern (Figure 5a(C) and 5b(C)) with bright circular rings corresponding to Bragg's reflection planes of (111), (200), (220), and (311) [40]. Thus, the SAED pattern also supported the XRD pattern of the present study.

### 3.6. Antibacterial Activity, MIC, and MBC Values of Ag NPs

The Ag NPs exhibited good antibacterial property, whereas the Au NPs did not show any inhibitory activity against the marine biofilm forming bacterial strains. Hence, the Ag NPs were screened for further investigation. The zone of inhibition against the marine biofilm forming bacterial strains ranged between 12.3 ± 0.47 and 15.6 ± 0.42 mm (Table 1). The maximum zone of inhibition (15.6 ± 0.42 mm) was registered against *E. coli*, followed by *Salmonella* sp. (14.5 ± 0.41 mm) > *S. liquefaciens* (13.2 ± 0.62 mm) > *A. hydrophila* (12.3 ± 0.47 mm). However, the macrotube dilution method inferred the MIC (20–40 *μ*L mL^−1^) and MBC (40–60 *μ*L mL^−1^) value of the Ag NPs (Table 1). The least inhibitory concentration (20 *μ*L mL^−1^) was recorded against *E. coli* and *Salmonella* sp., whereas the highest bactericidal concentration (60 *μ*L mL^−1^) was reported to inhibit all the bacterial strains tested. The *T. conoides* mediated Ag NPs are thus proved to reduce the bacterial densities significantly even at the least concentration. Sondi and Salopek-Sondi [41] reported that the Ag NPs disrupt the bacterial cells by alteration of the permeability of the cell membrane and finally cause the cell death. The size and shape of the nanoparticles play an important role in many pharmaceutical applications. The rapidly synthesised Ag NPs was identified between 2 and 17 nm with spherical shape, which gives better results compared with the Ag NPs synthesized using other plant materials [42], mangroves [43], seaweeds [27, 28], and coastal bacterial strains [44]. The present study supports that the Ag NPs synthesized using aqueous extract of *T. conoides* was found to be a potent source of antifoulant inhibiting the growth of marine biofilm forming bacteria.

### 3.7. Confocal Laser Scanning Microscopic Analysis

**Figure 6:**
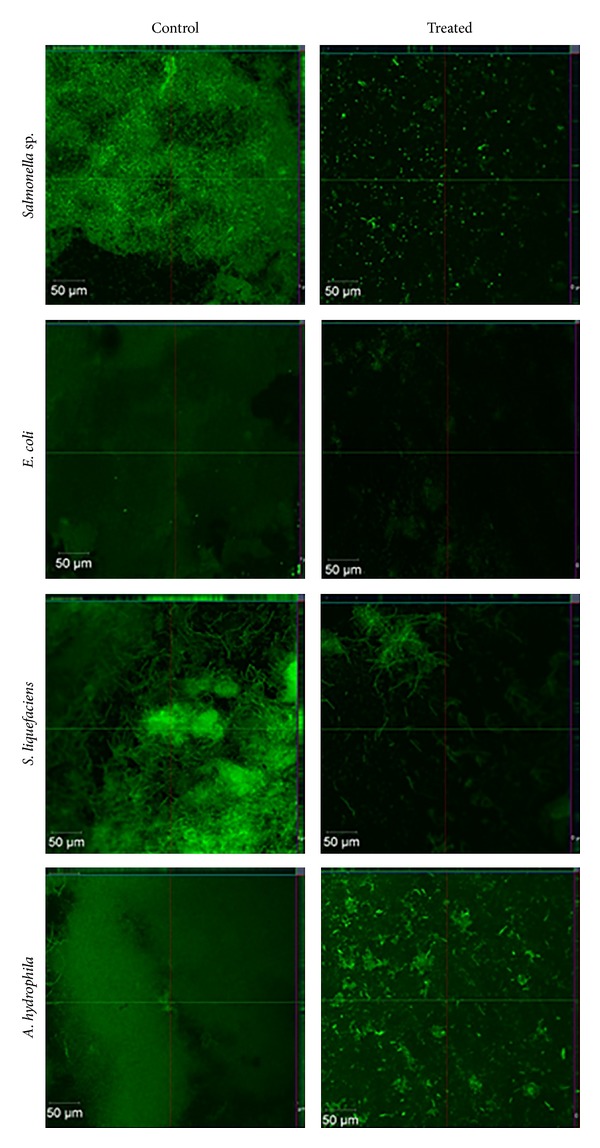
CLSM images representing the antibiofilm activity of Ag NPs against marine biofilm forming isolates of *Salmonella* sp., *E. coli*, *S. liquefaciens*, and *A. hydrophila*.

At 24 h of incubation, the CLSM image (Figure 6) depicted the weak adherence and disintegrated biofilm architecture of the test bacterial strains, that is, *Salmonella *sp., *E. coli*,* S. liquefaciens*, and *A. hydrophila*. In control, strong adhering ability of marine biofilm bacterial strains which led to the development of dense biofilm formation on the glass pieces was observed. Thus, the CLSM study confirmed that a substantial concentration of the Ag NPs were active in controlling the adherence and biofilm formation [45]. Prabhawathi et al. [19] also reported that the greener synthesis of protein stabilized Ag NPs coated on the surface of the polycaprolactam (polymer) proved to be a good antifouling agent against bacterial and fungal pathogens. The same technique was adopted earlier to measure the biofilm inhibition and adherences against *Streptococcus pyogenes* [46], *Staphylococcus aureus* [47, 48], and *E. coli* PHL628 [49] using different bacterial extracts. The nanoparticles precoated on the surfaces, surfactants, and enzymes inhibited the bacterial biofilm formation [50, 51]. Therefore, CLSM observations supported the antibiofilm property of Ag NPs strongly as one of the potent antifouling agents against marine biofilm formation under *in vitro* conditions.

### 3.8. Cytotoxicity and Anticrustacean Properties of Ag NPs

**Figure 7:**
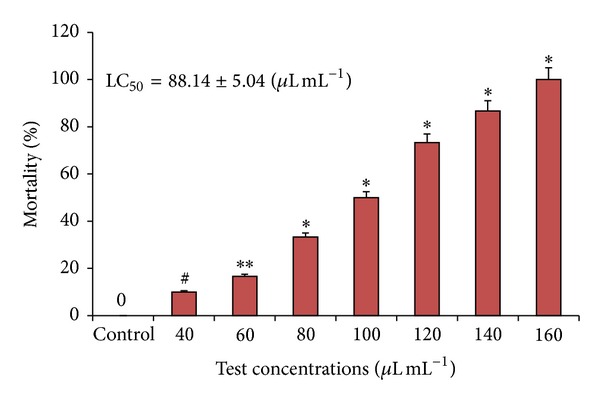
Cytotoxicity of Ag NPs on *Artemia salina*: increased concentration of Ag NPs (0–160 *μ*L/mL) (*X*-axis) mortality of the larvae up to 100% (*Y*-axis); all the data were expressed in mean ± SD of three replicates. *P < 0.0001 and **P < 0.01 indicate statistical significance; ^#^NS (not significant).

The *Artemia* cytotoxicity and anticrustacean assays are one of the reliable methods to screen and detect the cytotoxicity of the products. The LC_50_ values on the cytotoxicity of the synthesized colloidal Ag NPs was 88.91 ± 5.04 *μ*L mL^−1^, whereas the highest mortality was observed at 160 *μ*L mL^−1^ concentration. Lethality was found to be directly proportional to the concentration of Ag NPs (*P* < 0.01) (Figure 7). In accordance with the present study, Kumar et al. [52] have reported 100% mortality of *A. salina* at 100 nM concentration of the Ag NPs synthesized by *Sargassum ilicifolium*. Therefore, the biosynthesized Ag NPs can definitely be used as an ecofriendly antibiofilm agent against marine biofilm forming microorganisms and to prevent further succession. Interestingly, this is the first report on the antimicrofouling activity of the biologically synthesized Ag NPs using *T. conoides* against marine biofoulers.

## 4. Conclusion

The aqueous extract of seaweed *T. conoides* can be used as an effective reducing and capping agent for the single-pot biosynthesis of the Ag and Au NPs. The biologically synthesized Ag NPs with smaller size and outstanding nanostructures are promising materials against the formation of marine biofilms by bacteria and effectively lethal to brine shrimp *Artemia salina*. Hence, the present study suggests that the biologically synthesized clean, nontoxic, and environmentally acceptable nanoparticles can be applied as coating materials on the surfaces of the aquaculture installations and other marine systems to prevent the settlement and growth of the fouling consortia. Furthermore, *in vivo* studies are necessary to be carried out on these innovative antifouling strategies to validate the advantages of the synthesized Ag NPs.

## Figures and Tables

**Table 1:** *In vitro* antibacterial potential of biosynthesized Ag NPs using *T. conoides* extract against marine biofilm forming bacterial isolates.

Test organisms	Agar well diffusion assay (mm)	MIC (µL mL^−1^)	MBC (µL mL^−1^)
*Salmonella *sp.	14.5 ± 0.41	20	40
*E. coli *	15.6 ± 0.42	20	40
*S. liquefaciens *	13.2 ± 0.62	40	60
*A. hydrophila *	12.3 ± 0.47	40	60

Values ± SD indicate the three individual experiments.
